# Diaphragm microcirculatory dysfunction and lipid accumulation in endotoxemic rabbits during mechanical ventilation

**DOI:** 10.1186/cc13479

**Published:** 2014-03-17

**Authors:** Y Yang, T Yu, J Liu, C Pan, F Longhini, L Liu, Y Huang, F Guo, H Qiu

**Affiliations:** 1 Zhong-Da Hospital, Southeast University, Nanjing, China; 2 Eastern Piedmont University 'A. Avogadro, Novara, Italy

## Introduction

Sepsis-induced diaphragm dysfunction (SIDD) has been widely described in the literature as a condition affecting the diaphragm muscle characterized by contractility loss of function and associated with a high mortality, assessed at around 54% [1]. Previous studies have investigated the pathogenesis of ventilator-induced diaphragmatic dysfunction (VIDD), its lipid metabolic alterations [2] and microcirculatory function processes. This study was designed to investigate on diaphragm muscle the effects of LPS-induced endotoxemia in rabbits undergoing two different modes of mechanical ventilation.

## Methods

A prospective randomized animal study in 25 invasively monitored and mechanically ventilated New Zealand White rabbits. The rabbits were randomized to control (*n *= 5), controlled mechanical ventilation (CMV) (*n *= 5), pressure support ventilation (PSV) (*n *= 5), or CMV or PSV with LPS-induced endotoxemia (CMV-LPS and PSV- LPS respectively) (*n *= 5 for each). The endotoxemia was induced by LPS injection in the CMV-LPS and PSV-LPS groups. Rabbits were anesthetized and ventilated for 24 hours, except for the control (30 minutes). A catheter able to detect the electrical activity of the diaphragm was placed to evaluate the diaphragm contractility at baseline and after 6, 12 and 24 hours. After 24 hours, we evaluated: the diaphragm microcirculation assessed by a sidestream dark-field videomicroscopy; the mitochondria membrane potential; the lipid accumulation; and the diaphragm muscular fiber structure.

## Results

In endotoxemic animals, after 24 hours, the diaphragm contractility and fiber structure, the microcirculation, mitochondrial membrane potential and lipid accumulation were severely compromised, but not in the CMV and PSV groups. Moreover, a slight but significant increase of lipid accumulation was observed in the CMV and PSV groups in comparison with control (*P *< 0.05).

## Conclusion

In endotoxemic rabbits, the impaired microcirculation resulted in an increased lipid accumulation and in a disturbance of the mitochondria membrane potential and contractility of the diaphragm. No microvascular alterations have been observed in ventilated non-endotoxemic animals. Moreover, the diaphragm contractility dysfunction was more pronounced in endotoxemic animals.
